# Tolerability of PSMA radioligand therapy in metastatic prostate cancer patients with baseline mild to moderate leukopenia

**DOI:** 10.1186/s13550-025-01280-0

**Published:** 2025-07-06

**Authors:** Moritz B. Bastian, Tilman Speicher, Arne Blickle, Caroline Burgard, Julius L. D. Bastian, Mark Bartholomä, Andrea Schaefer-Schuler, Stephan Maus, Samer Ezziddin, Florian Rosar

**Affiliations:** 1 Department of Nuclear Medicine, Saarland University, Kirrberger Str., Geb. 50, 66421 Homburg, Germany; 2 Department of Urology, Saarland University, Homburg, Germany

**Keywords:** Leukopenia, PSMA, Radioligand therapy, RLT, mCRPC, Safety

## Abstract

**Background:**

Aim of this study was to analyze the safety of prostate-specific membrane antigen radioligand therapy (PSMA-RLT) in patients with metastatic castration-resistant prostate cancer (mCRPC) with preexisting mild to moderate leukopenia (CTCAE ≥ 1).

**Results:**

Thirty-seven mCRPC patients with preexisting leukopenia (leukocyte count < 3.8 × 10^9^/L) were included in this study. Patients received a median of 3 cycles of [^177^Lu]Lu-PSMA-617 (range 1–9). No significant difference in leukocyte counts was observed between baseline and follow-up after each PSMA-RLT cycle: first cycle (3.0 ± 0.5 at baseline vs. 3.4 ± 1.4 at follow up [in × 10^9^/L], *p* = 0.0921), second cycle (3.1 ± 0.4 vs. 3.8 ± 1.7, *p* = 0. 0.0509), and third cycle (3.1 ± 0.4 vs. 3.2 ± 2.0, *p* = 0.2929), respectively. Similarly, baseline and end of treatment values, irrespective of the number of administered cycles, did not reveal a significant difference (3.0 ± 0.5 vs. 3.5 ± 1.4, *p* = 0.0684). After the end of therapy, irrespective of the number of administered cycles, 27% patients remained stable in terms of CTCAE scoring, 46% changed to a higher score and 27% improved to a lower score.

**Conclusion:**

Although marked preexisting leukopenia is often considered a relative contraindication for PSMA-RLT, our findings indicate that PSMA-RLT is feasible in patients with leukopenia of CTCAE grade ≥ 1. In our cohort, leukocyte counts remained stable without significant RLT-induced deterioration. Therefore, patients with leukopenia should not be categorically excluded from receiving PSMA-RLT.

*Trial registration*: Clinicaltrials.gov identifier: NCT04833517, registered 01.01.2016.

**Supplementary Information:**

The online version contains supplementary material available at 10.1186/s13550-025-01280-0.

## Background

Prostate cancer ranks as the second most frequently diagnosed malignancy worldwide [1]. A significant proportion of cases advance to metastatic castration-resistant prostate cancer (mCRPC), a stage characterized by poor clinical outcomes [2–4]. While treatment options such as novel androgen axis drugs (NAAD) [5, 6], taxane-based chemotherapy [7, 8], radium-223 therapy [9], and PARP inhibitors [10, 11] exist, radioligand therapy (RLT) targeting prostate-specific membrane antigen (PSMA) has gained prominence as an effective alternative approach for mCRPC management [12–21]. PSMA, highly expressed on mCRPC cells [12, 13], serves as an ideal target for RLT. Despite its generally favorable safety profile, PSMA-RLT has been linked to hematologic toxicities, although significantly lower than chemotherapy. The VISION trial, for example, reported leukopenia in 12.5% of patients treated with [^177^Lu]Lu-PSMA-617 [20]. As a result, current guidelines issued by the EANM/SNMMI classify myelosuppression—among others preexisting leukopenia—as a relative contraindication for this therapy [22]. In a previous study, our group demonstrated that PSMA-RLT can be safely administered in patients with preexisting thrombocytopenia [23]. This study aims to assess the safety and hematologic effects of PSMA-RLT in individuals with preexisting leukopenia, particularly focusing on leukocyte count variations and therapeutic outcomes.

## Methods

In this study n = 37 mCRPC patients with pre-existing leukopenia, receiving PSMA-RLT were included in this study. Leukopenia was defined as leukocyte count < 3.8 × 10^9^/L, equaling a score ≥ 1, according to the ‘*common terminology criteria of adverse events*’ (CTCAE v5.0) and the in-house lower limit of normal (LLN) for leukocyte count, LLN = 3.8 × 10^9^/L. Identification was based on screening for leukopenia within the prospective registry titled ‘Prospective REgistry of Targeted RadionucLide TherapY in Patients With mCRPC (REALITY Study) (NCT04833517, https://clinicaltrials.gov/study/NCT04833517, registered 01.01.2016). All participants had previously received multiple treatments, including androgen deprivation therapy (ADT), NAAD, and taxane-based chemotherapy, prior to initiating PSMA-RLT. Detailed patient characteristics and treatment history are summarized in Table 1. PSMA-RLT was provided under compassionate use according to §13 (2b) of the German Pharmaceutical Act. Written informed consent was obtained from each participant after a thorough clarification of potential risks and side effects. Additionally, all patients consented to data publication in line with the Declaration of Helsinki, and the study received approval from the local ethics committee (approval number 140/17).

**Table 1:** Patient characteristics

Patient characteristics	Value
*Age*	
Median in [years], (range)	67 (47–91)
Age ≥ 65 years, n (%)	21 (55.3)
Age < 65 years, n (%)	17 (44.7)
*ALP, in [U/L]*	
Median (range)	183 (24–2064)
*Hemoglobin, in [g/dL]*	
Median (range)	10 (5–14)
< 13.5 g/dL, n (%)	33 (92)
*ECOG performance status, n (%)*	
0	7 (18.9)
1	11 (29.7)
≥ 2	19 (51.4)
*Prior therapies, n (%)*	
Prostatectomy	12 (32.4)
Radiation	15 (40.5)
ADT	37 (100)
NAAD	32 (86.5)
Abiraterone	24 (64.9)
Enzalutamide	29 (78.4)
Abiraterone and Enzalutamide	15 (40.5)
Chemotherapy	27 (73.0)
1st line Docetaxel	27 (73.0)
2nd line Cabazitaxel	14 (37.8)
Time since last therapy (median months)	7.4 (range 1–35.1)
[^223^Ra]Ra-dichloride	12 (32.4)
Time since last therapy (median months)	2.1 (range 1–52.1)
Other	1 (2.7)
*PSA at baseline, in [ng/mL]*	
Median (range)	131 (2–3277)
*Metastases*	
Bone	36 (97.3)
LN	26 (70.3)
Liver	8 (21.6)
Lung	5 (13.5)
Other (cerebral, spinal, other visceral)	3 (0.8)

ADT, androgen deprivation therapy; ALP, alkaline phosphatase; ECOG, eastern cooperative oncology group; NAAD, novel androgen axis drugs; PSA, prostate specific antigen

### Treatment details

The radiolabeling and the quality control were performed according to the established standard procedures [22, 24]. Patients received a median of 3 cycles PSMA-RLT (range 1–9) with a median time interval of 6 weeks between consecutive cycles of [^177^Lu]Lu-PSMA-617. The mean administered activity of [^177^Lu]Lu-PSMA-617 per cycle was 6.8 ± 1.5 GBq (2.7–10.2 GBq) and the mean cumulative activity was 25.4 ± 16.3 GBq (2.7–62.0 GBq), respectively. The administered activities were adjusted individually, as previously introduced by Khreish et al. [15]. In addition, 16 patients received 1–4 [^225^Ac]Ac-PSMA-617- augmented cycles (in total 31 cycles) within [^177^Lu]Lu-PSMA-617 RLT, with a mean activity of 3.6 ± 2.1 MBq (range: 0.8–10.1 MBq) per cycle with a mean cumulative [^225^Ac]Ac-PSMA-617 activity of 7.0 ± 5.6 MBq (range: 1.3–19.3 MBq). Augmentation with [^225^Ac]Ac-PSMA-617 was initiated in patients who showed insufficient response to prior [^177^Lu]Lu-PSMA-617 monotherapy, as determined by serial PSA measurements, imaging assessments, and clinical evaluation. All radioligand administrations complied with German radiation protection regulations and were performed during inpatient stays. Patients received intravenous hydration with 500 mL of 0.9% NaCl and salivary gland cooling, starting 30 min prior to infusion. Radioligand infusions were administered intravenously over a 1-h period via an infusion line.

### Monitoring and statistics

The course of leukocyte cell count was closely monitored within and after the PSMA-RLT and analyzed statistically and according to CTCAE, with baseline laboratory tests < 24 h before administration of the first PSMA-RLT cycle and subsequent frequent blood sampling either in-house or at the referring physician’s office (general practitioner, urologist or oncologist). Additional parameters, including platelet count, hemoglobin levels, and glomerular filtration rate (GFR), were also assessed. For statistical analysis, descriptive analysis, Shapiro–Wilk test and the paired t-test was used for comparisons using Prism 8 (GraphPad Software, San Diego, CA, USA), to evaluate possible differences in leukocyte counts between baseline and follow-up examinations. A *p*-value < 0.05 was defined as statistically significant.

### Response and outcome

Treatment response was monitored through biochemical analysis, specifically tracking changes in serum PSA levels. Based on these measurements, outcomes were classified into three categories: partial remission (PR), stable disease (SD), or progressive disease (PD). In accordance with the guidelines set by the Prostate Cancer Working Group 3 (PCWG3) [25], PD was defined as a serum PSA increase of 25% or more from baseline values. A PSA reduction of at least 50% was considered indicative of PR, while SD included cases where PSA levels decreased by less than 50% or increased by less than 25%. Progression-free survival (PFS) was determined from the initiation of RLT to the point of disease progression (PSA increase exceeding 25%), death, or the final recorded study visit. Overall survival (OS) was calculated from the beginning of RLT to the date of death or the last follow-up visit. The follow-up data used in this study were current as of March 15, 2025.

## Results

### Response and survival

**Fig. 1:**
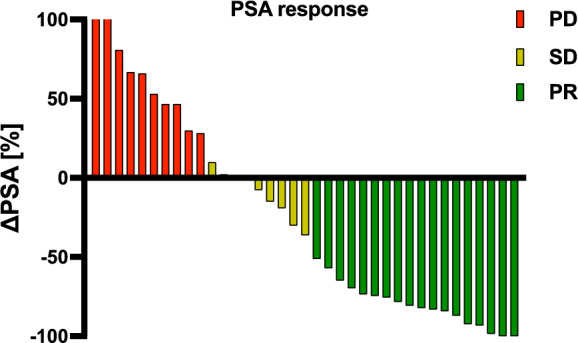
Waterfallplot of individual change in PSA (ΔPSA) for each patient with classification in partial remission (PR), stable disease (SD) and progressive disease (PD)

**Fig. 2:**
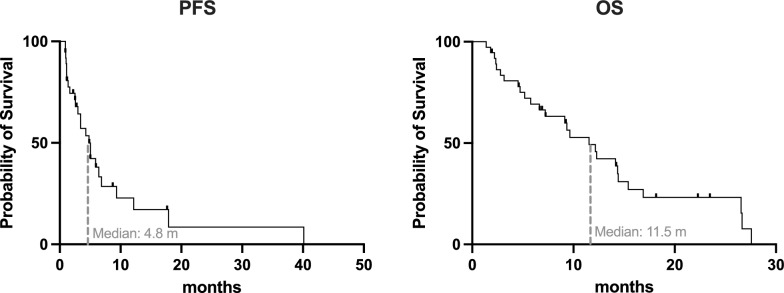
Kaplan–Meier curves presenting the PSA based progression-free survival (PFS) and the overall survival (OS) of patients

Response and survival assessment demonstrated that the majority of patients experienced either a PR (18/37 patients; 48.7%) or SD (9/37 patients; 24.3%) after PSMA-RLT. The remaining 10 patients showed PD, representing 27% of the cohort. The mean change of PSA considering all patients was—36.2 ± 72.0%. The individual PSA response is presented in Fig. 1. As presented in Fig. 2 the survival analysis by Kaplan–Meier method showed a median PFS of 4.8 months (CI: 2.9–6.8) and a median OS of 11.5 months (CI: 7.8–15.2), respectively.

### Leukocyte analysis

**Fig. 3:**
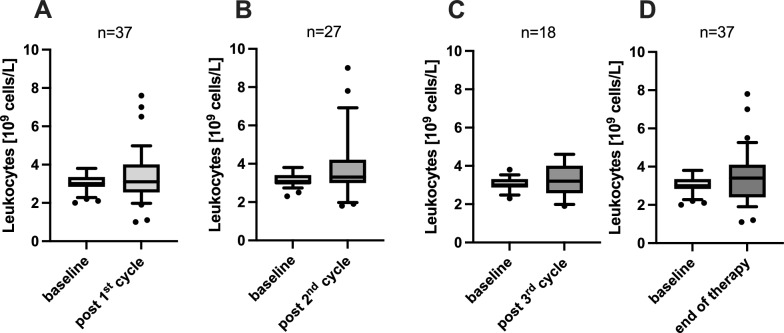
Comparison of the leukocyte cell number referring to baseline, juxtaposing the cell count after the first (**A**), the second (**B**) and the third cycle of [^177^Lu]Lu-PSMA-617 (**C**), as well as after the end of therapy (**D**)

**Fig. 4:**
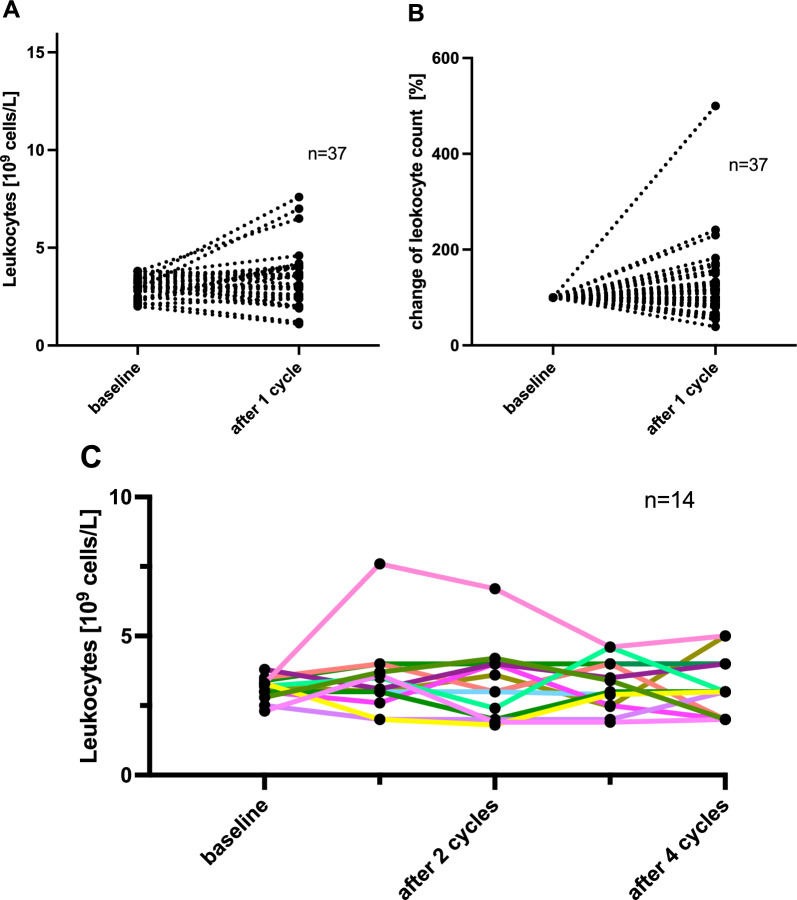
Absolute (**A**) and relative (**B**) changes in leukocyte counts for all patients (n = 37). Development of leukocyte cell count for each individual patient receiving four cycles of PSMA-RLT (**C**), (n = 14)

The analysis of the leukocyte cell count demonstrated that the number of leukocytes remained stable during the PSMA-RLT. At baseline, a mean cell count of 3.0 ± 0.5 × 10^9^/L was assessed. After one cycle of [^177^Lu]Lu-PSMA-617, this value showed no statistical difference, with a mean cell count of 3.4 ± 1.4 (*p* = 0.0921). Following a second cycle of PSMA-RLT the number leukocytes presented also no significant difference of the mean from 3.1 ± 0.4 to 3.8 ± 1.7 (*p* = 0.0509). After a third cycle of [^177^Lu]Lu-PSMA-617 was administered a mean leukocyte count of 3.2 ± 2.0 (vs. 3.1 ± 0.4 at baseline) was evaluated, presenting no significant change compared to baseline (*p* = 0.2929). Similarly, a comparison of baseline and end of therapy values, independent from the number of administered cycles, showed a stable number of leukocytes after PSMA-RLT, with a mean cell count of 3.5 ± 1.4, which presents no significant difference if juxtaposed to baseline (*p* = 0.0684). The respective comparisons of leukocyte cell count during application of PSMA-RLT are visualized in Fig. 3. The absolute and relative changes in leukocyte counts for all patients (n = 37) are illustrated in Fig. 4a and b. The development of leukocyte cell counts for each individual patient receiving four cycles of PSMA-RLT (n = 14) is presented in Fig. 4c, indicating a stable trend over the entire time-span, for the majority of patients.

### CTCAE leukocyte grading

**Fig. 5:**
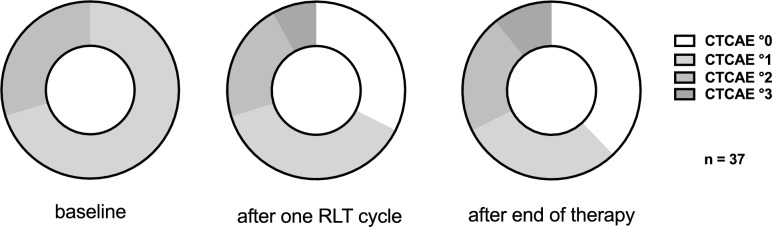
The CTCAE grading addressing leukocytopenia at baseline, after one cycle of [^177^Lu]Lu-PSMA-617 and after end of therapy

**Fig. 6:**
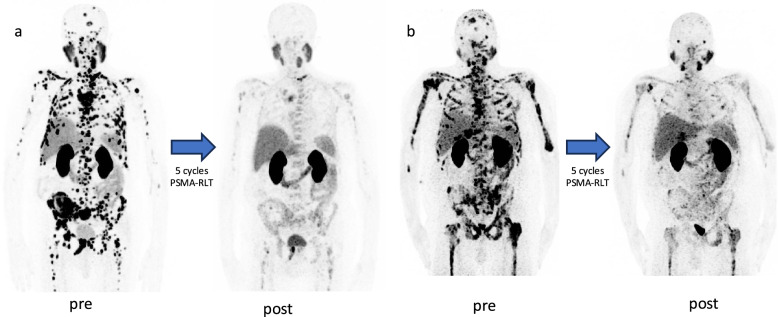
Maximum intensity projections (MIP) of [^68^ Ga]Ga-PMSA-11 PET/CT images, presenting two patients before and after five cycles of [^177^Lu]Lu-PSMA-617

The grading of leukocytopenia following the CTCAE criteria showed that 14 patients experienced an improvement of the condition following the first cycle of PSMA-RLT, while 16 patients presented a stable scoring and 7 a worsening. Similarly, comparing baseline and end of therapy 17 (46%) patients presented an improved CTCAE score, 10 (27%) patients with stable leukocytopenia condition and 10 (27%) patients experiencing a worsening of the grading. The course of CTCAE gradings addressing leukocytopenia is depicted in Fig. 5. No CTCAE score over 3 was recorded for leukocytopenia. Figure 6 presents two patients with clearly notable response to PSMA-RLT, following 5 cycles of [^177^Lu]Lu-PSMA-617.

### Further hematologic parameters

Considering further hematological parameters, the mean hemoglobin level prior to the initiation of RLT was 10.3 ± 2.1 g/dL. Following RLT, a slight decrease in hemoglobin levels was observed, with a post-treatment mean of 9.7 ± 2.0 g/dL. While 30 patients exhibited anemia before treatment, nine individuals experienced a further decline in their hemoglobin levels over the course of therapy, whilst 6 patients showed an improvement according to CTCAE (see supplement Table S1).

Platelet counts also showed a slight reduction following RLT. Before treatment, the mean platelet count was 153.8 ± 71.8 × 10^9^/L. Following RLT, platelet counts slightly decreased, with a mean value of 125.6 ± 74.7 × 10^9^/L. Prior to RLT, seventeen patients had thrombocytopenia, whereas post-treatment, five patients showed newly developed thrombocytopenia whilst two patients showed an improvement to grade 0 according to CTCAE (see supplement Table S2).

Two patients discontinued treatment due to pancytopenia and severe deterioration in the patient’s general condition.

In addition to hematological parameters, renal function was assessed using the GFR. Prior to RLT, the mean GFR was 90.5 ± 14.5 mL/min/1.73 m^2^. Following therapy, a post-treatment mean GFR of 89.2 ± 16.9 mL/min/1.73 m^2^ was observed. The majority of patients showed no renal impairment during the course of therapy. Thirteen patients showed renal impairment prior to treatment, 3 of these patients experienced a mild decline in CTCAE grading, and 2 patients experienced an improvement of CTCAE grading (see supplement Table S3).

### Augmented subgroup

For the 16 patients receiving 1–4 [^225^Ac]Ac-PSMA-617-augmented cycles (a total of 31 cycles, with a median of 2), leukocyte levels remained stable, with end-of-therapy values showing no significant difference compared to baseline (mean cell count: 3.13 ± 0.42 vs. 3.24 ± 0.97; *p* = 0.6740). According to CTCAE criteria, pre-therapy leukocytopenia grading revealed that 15 patients had grade 1 and 1 patient grade 2; following PSMA-RLT, 8 patients showed improvement, 4 remained stable, and 4 worsened. With post-therapy CTCAE grading of 7 grade 0, 5 grade 1 and 4 grade 2, respectively. Regarding other hematological parameters, mean hemoglobin levels stayed stable with 10.36 ± 1.88 g/dL before treatment to 10.0 ± 1.61 g/dL after RLT; while 16 patients were anemic at baseline, 5 experienced further decline, and 2 showed improvement as per CTCAE (see Supplement Table S4). Platelet counts declined slightly post-RLT, from a mean of 154.1 ± 54.71 × 10^9^/L at baseline to 127.4 ± 75.44 × 10^9^/L; 10 patients had thrombocytopenia before RLT, with 3 developing new thrombocytopenia (grade 1) and 1 showing improvement to grade 0 after therapy (see Supplement Table S5). In addition to hematological changes, renal function assessed via GFR remained largely unaffected, with pre-treatment mean GFR at 91.13 ± 14.64 mL/min/1.73 m^2^ and post-treatment mean at 86.78 ± 14.62 mL/min/1.73 m^2^; most patients experienced no renal impairment, though 6 had pre-existing impairment, among whom 1 showed mild CTCAE grade decline from grade 1 to 2 and one patient showed newly grade 1 impairment (from 92 to 88 mL/min/1.73 m^2^, see Supplement Table S6).

## Discussion

This study demonstrates that PSMA-RLT is both safe and effective in mCRPC patients with preexisting leukopenia. Leukocytopenia prior to RLT may result from several factors, including prior chemotherapy with myelosuppressive agents such as taxanes [7, 26], extensive bone marrow involvement by metastatic disease[27–29], and systemic inflammation associated with advanced cancer, which can impair hematopoiesis through cytokine-mediated suppression [30, 31]. These mechanisms may compromise baseline bone marrow function, potentially impacting treatment tolerability and hematologic outcomes. However, contrary to concerns regarding further leukocyte depletion, our findings indicate that leukocyte counts remained largely stable throughout the treatment course, with no significant decline observed between baseline and follow-up assessments. Moreover, a substantial proportion of patients achieved either partial remission or stable disease, underscoring the therapeutic potential of PSMA-RLT in this compromised cohort. Two patients discontinued treatment due to pancytopenia and severe deterioration in the patient’s general condition.

When comparing our findings to existing literature, the hematologic safety profile observed in our cohort aligns with previous reports from trials evaluating PSMA-RLT in broader mCRPC populations [15, 18–21]. The VISION trial by Sartor et al., for example, documented leukopenia rates of 12.5% among patients receiving [^177^Lu]Lu-PSMA-617, though it excluded individuals with preexisting leukopenia under a leukocyte cell count of 2.5 × 10^9^/L [20]. Our data extend these findings by demonstrating that even in a patient group with already diminished leukocyte counts, PSMA-RLT does not significantly exacerbate hematologic toxicity. The survival outcomes observed in our cohort are also consistent with those reported in the literature from various prospective and retrospective studies [15, 18–21]. For instance, the VISION trial, reported PFS of 8.7 months and an OS of 15.3 months [20]. In contrast, while chemotherapy remains a cornerstone in mCRPC treatment, it is frequently associated with hematologic toxicity, including leukocyte depletion [32]. In patients with preexisting leukopenia, this can trigger a cascade of complications, including an increased risk of nosocomial infections, rendering chemotherapy a less suitable or even contraindicated option in this subgroup. In this context, PSMA-RLT stands as a valuable therapeutic alternative for patients traditionally considered ineligible for systemic therapies due to compromised hematopoiesis.

In comparison, the TheraP trial, required a neutrophil count ≥ 1.5 × 10^9^/L for inclusion, thereby selecting a population with at least mild neutropenia (CTCAE Grade 1) but excluding those with more severe hematologic compromise [18]. Since neutrophils represent the largest subset of leukocytes and play a key role in immune defense, this criterion ensured that patients in TheraP had adequate infection-fighting capacity. Our study, however, included patients with leukopenia, reflecting a broader reduction in all white blood cell types, including neutrophils, lymphocytes, and monocytes. Future studies are warranted to investigate the differential effects of PSMA-RLT on leukocyte subsets, particularly in patients presenting with marked neutropenia, lymphopenia, or monocytopenia, and to determine the clinical implications of these hematologic profiles. Notably, clinically relevant infectious complications in patients with leukopenia during systemic therapies may include exemplary pneumonia, urinary tract infections, sepsis, or fungal infections. Particularly in neutropenic patients, these infections can develop rapidly and pose a significant clinical risk. Although our cohort did not show a notable incidence of such complications during PSMA-RLT, patients with marked leukocyte depletion should be monitored for signs of infection throughout the treatment course.

Beyond leukocytes as hematologic parameter, our study also assessed additional safety aspects, including hemoglobin and platelet counts, which exhibited only minor declines over the course of treatment. These findings mirror prior investigations suggesting that bone marrow toxicity following PSMA-RLT is generally mild and manageable [23]. Moreover, renal function remained stable in most patients, further underscoring the favorable safety profile of this treatment. It is worth noting that, even in cases where [^225^Ac]Ac-PSMA-617-augmented therapy was administered, no increase in clinically relevant toxicity was observed, further underscoring the favorable safety profile of this approach even in intensified treatment regimens. Notably, inflammatory responses could play a role in individual variations of leukocyte kinetics, though our data do not suggest a clinically significant impact in this context. Future studies incorporating inflammatory biomarkers may provide deeper insights into potential interactions between immune responses and PSMA-RLT.

Despite these encouraging results, our study is subject to certain limitations. The relatively small cohort size and single-center nature of our investigation may limit the generalizability of our findings. Additionally, while our prospective registry provides robust real-world data, the absence of a direct control group prevents definitive conclusions regarding comparative hematologic effects. A further possible limitation of this study lies in the time gap between previous treatments (chemotherapy or ^223^Ra therapy) and the start of PSMA-RLT, with median intervals of 7.4 and 2.1 months, and minimum intervals of 1 month each, respectively. These treatments may have short-term effects on bone marrow function, possibly enabling leukocyte counts to recover during this period, which could introduce bias into the results. Furthermore, 17 individuals included in the analysis received [^225^Ac]Ac-PSMA-617 as part of an alpha-augmented RLT regimen, which may have influenced the overall outcomes. The long-term effects of PSMA-RLT on hematologic parameters were not assessed in this study; future research should aim to evaluate delayed toxicities and sustained hematologic recovery in patients with preexisting leukopenia. Further multicenter studies with larger cohorts and randomized designs would be valuable in confirming our results and establishing standardized treatment guidelines for patients with preexisting leukopenia. Furthermore, a healthy survivor bias cannot be ruled out, as patients receiving multiple cycles of PSMA-RLT may represent a more resilient subgroup, which could influence the interpretation of safety and outcome data.

## Conclusion

In conclusion, our findings provide important evidence that PSMA-RLT is a safe and effective therapeutic option for patients with mCRPC and preexisting leukopenia. Despite baseline hematologic compromise, patients tolerated treatment well, with stable leukocyte counts and no significant hematologic toxicity observed. The favorable safety and efficacy outcomes highlight the potential of PSMA-RLT to serve as a viable therapy in individuals with limited bone marrow reserve.

## Supplementary Information

Supplementary file1.

## Data Availability

The datasets used and/or analysed during the current study are available from the corresponding author on reasonable request.

## Abbreviations

mCRPC: Metastatic castration-resistant prostate cancer
NAAD: Novel androgen axis drugs
RLT: Radioligand therapy
PSMA: Prostate-specific membrane antigen
CTCAE: Common terminology criteria of adverse events
LLN: Lower limit of normal
ADT: Androgen deprivation therapy
GFR: Glomerular filtration rate
PR: Partial remission
SD: Stable disease
PD: Progressive disease
PFS: Progression-free survival
OS: Overall survival
