# Supplementary material for: Tolerability of PSMA radioligand therapy in metastatic prostate cancer patients with baseline mild to moderate leukopenia
Source: EJNMMI Res. 2025 Jul 6;15:82. doi: 10.1186/s13550-025-01280-0 (PMC12229971; doi:10.1186/s13550-025-01280-0)
Supplement: Supplementary file 1 — Supplementary file1. [file 13550_2025_1280_MOESM1_ESM.docx]

**Supplementary table S1.** CTCAE v5.0 Anemia:

| CTCAE Grade | Pre-RLT | Post-RLT |
| --- | --- | --- |
| °0 | 3 | 1 |
| °1 | 17 | 16 |
| °2 | 13 | 15 |
| °3 | 4 | 5 |
| °4 | 0 | 0 |

**Supplementary table S2.** CTCAE v5.0 Thrombocytopenia

| CTCAE Grade | Pre-RLT | Post-RLT |
| --- | --- | --- |
| °0 | 21 | 16 |
| °1 | 9 | 12 |
| °2 | 3 | 1 |
| °3 | 3 | 5 |
| °4 | 1 | 3 |

**Supplementary table S3.** CTCAE v5.0 eGFR (estimated Glomerular

Filtration Rate):

| CTCAE Grade | Pre-RLT | Post-RLT |
| --- | --- | --- |
| °0 | 23 | 18 |
| °1 | 12 | 16 |
| °2 | 2 | 3 |
| °3 | 0 | 0 |
| °4 | 0 | 0 |

**Supplementary table S4.** CTCAE v5.0 Anemia for augmented-therapy subgroup:

| CTCAE Grade | Pre-RLT | Post-RLT |
| --- | --- | --- |
| °0 | 0 | 0 |
| °1 | 8 | 6 |
| °2 | 8 | 8 |
| °3 | 0 | 2 |
| °4 | 0 | 0 |

**Supplementary table S5.** CTCAE v5.0 Thrombocytopenia for augmented-therapy subgroup:

| CTCAE Grade | Pre-RLT | Post-RLT |
| --- | --- | --- |
| °0 | 9 | 6 |
| °1 | 2 | 6 |
| °2 | 3 | 0 |
| °3 | 1 | 3 |
| °4 | 1 | 1 |

**Supplementary table S6.** CTCAE v5.0 eGFR for augmented -therapy subgroup:

| CTCAE Grade | Pre-RLT | Post-RLT |
| --- | --- | --- |
| °0 | 10 | 9 |
| °1 | 5 | 5 |
| °2 | 1 | 2 |
| °3 | 0 | 0 |
| °4 | 0 | 0 |
